# Conjugated Polymers-Based Ternary Hybrid toward Unique Photophysical Properties

**DOI:** 10.3390/molecules27207011

**Published:** 2022-10-18

**Authors:** Bandar Ali Al-Asbahi, Mohamad S. AlSalhi, Mohammad Hafizuddin Hj. Jumali, Amanullah Fatehmulla, Saif M. H. Qaid, Wafa Musa Mujamammi, Hamid M. Ghaithan

**Affiliations:** 1 Department of Physics & Astronomy, College of Sciences, King Saud University, Riyadh 11451, Saudi Arabia; 2 Department of Physics, Faculty of Science, Sana’a University, Sana’a 12544, Yemen; 3 School of Applied Physics, Faculty of Science and Technology, Universiti Kebangsaan Malaysia, Bangi 43600, Malaysia; 4 Department of Physics, Faculty of Science, Ibb University, Ibb 70270, Yemen

**Keywords:** F8, MEH-PPV, OC1C10-PPV-DMP, optical and optoelectronic properties, OLED

## Abstract

The improvement of optical and optoelectronic properties of the individual poly [2-methoxy-5- (2-ethylhexyloxy)-1,4-phenylenevinylene] (MEH-PPV), poly[2-methoxy-5-(3,7-dimethyl-octyloxy)-1,4-phenylenevinylene]–End capped with Dimethyl phenyl (OC1C10–PPV–DMP), and poly (9,9′-di- n -octylfluorenyl-2,7-diyl) (F8) was revealed by blending them in ternary hybrid with optimal ratio (F8/2 wt.% MEH-PPV/2 wt.% OC1C10–PPV–DMP). All individual and optimal ternary solutions were prepared via the solution-blending method followed by depositing them onto glass and ITO substrates using spin-coating technique. The semi-crystalline phase of the ternary hybrid and the strong mixing between the conjugated polymers were evidenced by observing the X-ray diffraction patterns that related to F8 into the hybrid diffractogram. The optical and optoelectronic properties of all prepared thin films were investigated in terms of absorption and emission spectra, Commission International d′Eclairage (CIE) coordinates, and current–voltage (I-V) characterizations. Emission peaks at the entire range of visible spectrum can be revealed from the ternary hybrid of the three individual conjugated polymers, producing white emission as evidenced from the emission spectrum and CIE coordinates of the hybrid. Among all fabricated organic light-emitting diodes (OLEDs) devices, the ternary hybrid-based-OLED revealed the best performance in terms of current and turn-on voltage.

## 1. Introduction

The hybrids of conjugated polymers (CPs) are of increasing application in electronics and optoelectronics, for example, transistors, photoemission devices, sensors and solar cells [1,2,3,4]. Remarkably, mixing different CPs results in merging numerous different assets of diverse compounds in one material. Regarding photoemission devices, mixing CPs presented light tunability and polychrome emissions. As white light emission is characteristically accomplished by mingling the three foremost colors (red, green, and blue), at least two emissive materials are coated in a multilayer structure [5,6,7] or classified as a single layer by doping (or mixing) [8,9,10]. The existence of various fluorophores tips into one of the following intermolecular interactions in CPs mixing [11]: (i) Ground states interaction to form a new state identified as a ground-state complex, that affects the emission and absorption spectrum; (ii) Exciplex and excimer formation subsequently photo-excitation; in this situation the interaction occurs between ground state and the excited state of the molecules; (iii) Energy transfer amongst altered fluorophores. The fluorophore having the highest energy band-gap between the lowest unoccupied molecular orbital (LUMO) and the highest occupied molecular orbital (HOMO) is named donor and the other fluorophores are named acceptors.

The source of Förster resonance energy transfer (FRET) is a spectral overlay amid acceptor absorption and donor emission, space amid acceptor and donor, and the placing of the dipoles of donor and acceptor molecules and the dominant medium [12,13]. The FRET emerges due to dipole–dipole interaction, where the separation between acceptor and donor must be less than 100 Å [14,15]. Three different methods have been deliberated on to evaluate the Förster radius: spectral overlap [16]; photoluminescence quantum efficiency [17]; and a direct measurement of the energy transfer rate [18]. In all these methods, it is necessary to consider the molecules of the polymer as a solid sphere so that the concentration of a particular combination can be linked to the intermolecular space of the acceptor and the donor. In the measurements of steady-state photoluminescence, the energy transfer in most composites was perceived to be a two-step process involving exciton migration in the donor and then FRET from molecules of the donor to those of acceptor [19,20]. The technique of FRET was subjugated in the current work to harvest WOLED with enhanced outcome. In addition to the good spectral overlap between donor emission and acceptor absorption, the good blending between them is required to produce FRET excitation in the hybrid [21]. Mixing of both the donor and acceptor for FRET can significantly decrease the concentration quenching of the excitons created, improving the device performance [22,23,24].

In the present work, we considered the ternary blend of “poly (9,9′-di- n -octylfluorenyl-2,7-diyl) (F8)”, “poly [2-methoxy-5- (2-ethylhexyloxy)-1,4-phenylenevinylene] (MEH-PPV)” and “poly[2-methoxy-5-(3,7-dimethyl-octyloxy)-1,4-phenylenevinylene]–end capped with Dimethyl Phenyl (OC1C10–PPV–DMP)” to accomplish cascade energy transfer for regulating emission colors and enhancing device performance. Three diverse emission modules of F8/OC1C10-PPV-DMP/MEH-PPV with the energy-band gap order of F8 (3.0 eV) > OC1C10-PPV-DMP (2.2 eV) or MEH-PPV (2.2 eV) [25,26,27] were miscible with each other so that the energy levels of both MEH-PPV and OC1C10-PPV-DMP fall within that of F8 and thus the possibility of a FRET in this system. Additionally, the emission spectrum of the donor (F8) and the absorption spectrum of the acceptor (MEH-PPV or OC1C10-PPV-DMP) coincided considerably. The leading constituent of F8 acted as a matrix, diluent, excitation energy donor for the ternary hybrid and for generating light with high efficiency. Therefore, once the ternary hybrid was excited near the donor absorption peak wavelength, light emission can be predicted from both acceptors, suggesting the cascade energy transfer used for WOLEDs. As the studies on the photophysical mechanisms of ternary hybrid systems are exceptional, the present work concentrated on how the dual FRET mechanism can improve optoelectronic properties of the individual polymer and produce OLED device with desired white emissions and lower turn-on voltage.

## 2. Materials and Methods

The OC1C10-PPV-DMP (Mw~100,000 g/mol) was obtained from American Dye Source, Inc. (Morgan Boulevard, QC, Canada), whereas MEH-PPV (Mw~40,000 g/mol) and F8 (Mw~58,200 g/mol) were purchased from Sigma Aldrich (Saint Louis, MO, USA) and used as received without any purification. All of these conjugated polymers were dissolved separately in toluene prior to preparing optimal ternary blend by blending the F8 with 2.0 wt.% of each acceptor (OC1C10-PPV-DMP and MEH-PPV) using the blending solution method, as confirmed in our recent report where the emission of each component was in balance with each other [27]. The concentration of F8 was 15 mg/mL, whereas the concentration of each acceptor was 0.15 mg/mL in the ternary hybrid. All samples from the toluene solutions were separately spin-coated onto clean glass substrates, for absorption and emission spectra characterizations, and onto etched ITO substrates for OLED fabrications. A 50 μL of each sample was deposited onto the substrates by spin coating technique at 2000 rpm for 30 s, thus the obtained thickness of all thin films was close to 130–150 nm (130 nm for F8, 140 nm for MEH-PPV, 143 nm for OC1C10-PPV-DMP, and 150 nm for the ternary hybrid thin film). The structure of all prepared films was characterized by X-ray diffraction (XRD; Miniflex 600, Rigaku, Japan) with a Cu-Kα radiation source (λ = 1.5418 Å). The diffractograms were collected in the 2θ range of 5° to 80° with a step size of 0.025°.

**Figure 1:**
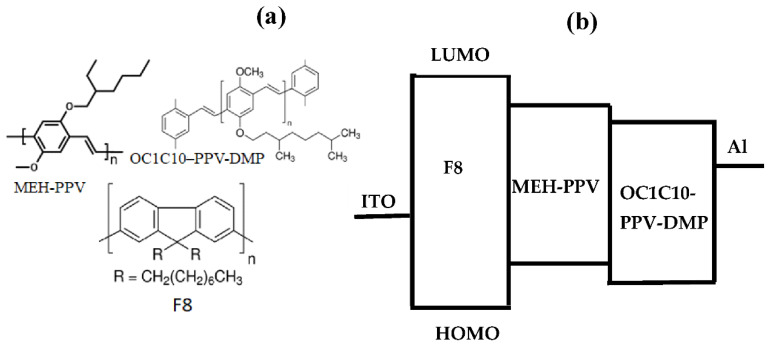
(**a**) Chemical structure of the F8, MEH-PPV and OC1C10-PPV-DMP; (**b**) Energy levels schematic of all used materials.

Both absorption and emission spectra were obtained using a UV-Vis spectrometer (JASCO V-670, Cremella, Italy) and spectrofluorometer (JASCO FP-8200, Cremella, Italy), respectively. The OriginLab program version 2019b (Northampton, MA, USA) was used to collect the CIE coordinates of all thin films from their emission data. For OLEDs fabrication, each coated ITO substrate was positioned into an electron-beam chamber for the aluminum cathode deposition with 150 nm thick at a deposition rate of 2 Å/min. A Keithley 238 measurement system was employed for current–voltage (I-V) and thus turn-on voltage. The chemical structure of the conjugated polymers and the energy levels schematic of all used materials are presented in Figure 1a,b, respectively.

## 3. Results and Discussion

**Figure 2:**
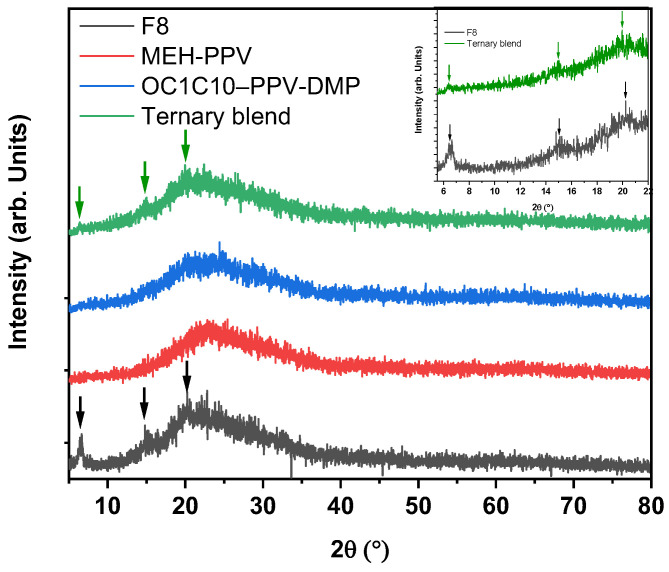
X-ray diffraction spectra of pristine F8, MEH-PPV, OC1C10-PPV-DMP and their ternary hybrid. The inset shows the narrow peaks in both F8 and ternary hybrid diffractograms.

Figure 2 shows the X-ray diffraction (XRD) patterns for pristine F8, MEH-PPV, OC1C10–PPV-DMP and their ternary hybrid. A broad peak in the range 2θ of 10.0°–40.0° was indicated for all thin films of conjugated polymers, indicating the amorphous phase of all thin films. Despite the dominant amorphous phase of F8, few narrow peaks with low intensity were marked by arrows at 6.5, 15.0 and 20.2°, indicating the semi-crystalline structure of the F8 [28,29]. As clearly shown inset in Figure 2, these narrow peaks can be also detected in the thin film of ternary hybrid, indicating the strong incorporation between the compositions and that the ternary hybrid has a semi-crystalline structure that is placed between the amorphous and crystalline phases. Since neither new peak appeared in the hybrid nor was there any shifting in the narrow peaks, there is no variation in the structure of the polymers due to the blending process being detected.

**Figure 3:**
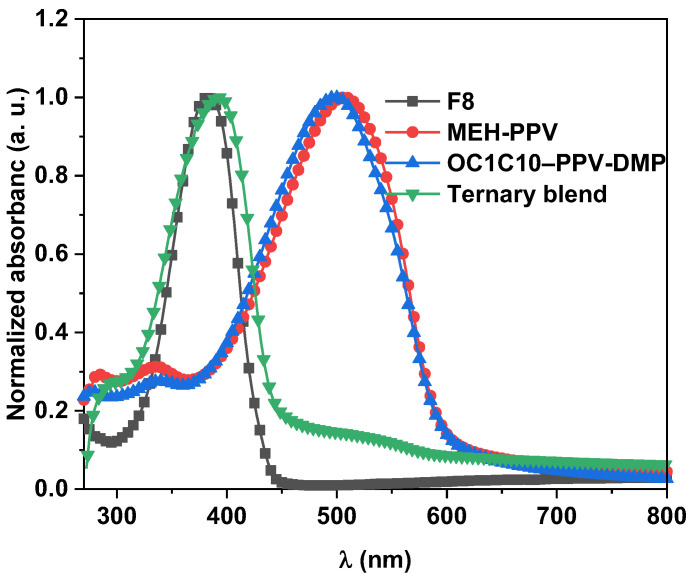
Normalized absorbance spectra of pristine F8, MEH-PPV, OC1C10–PPV-DMP and their ternary hybrid.

Figure 3 shows normalized absorption spectra of pristine thin films of F8, MEH-PPV, OC1C10-PPV-DMP and their optimal ternary hybrid. The maximum absorbance of F8, MEH-PPV and OC1C10-PPV-DMP was at 385, 507 and 500 nm, respectively. Both MEH-PPV and OC1C10-PPV-DMP have shoulders in the UV-region at 336 and 284 nm for MEH-PPV while they are at 337 and 276 nm for OC1C10-PPV-DMP. Upon incorporating both the acceptors into the donor to form the ternary blend, the main absorbance of F8 was red-shifted to 391 nm while the main absorbance of both acceptors was red-shifted to 512 nm and their shoulders to 294 nm. This finding may indicate the possibility of decreasing the optical band gap of the donor and thus increasing its conjugation length [24,30]. The cut-off wavelength of the donor absorbance edge also red-shifted from 444 nm to 450 nm in the ternary blend. The energy tail (E_tail_) of the donor, which refers the expansion of tail depth states into the forbidden energy gap below the absorption edge, was decreased from 2.793 to 2.755 eV in the ternary blend while the steepness parameter (σ), which indicates the extension/shrinkage of the optical absorption edge due to the exciton–phonon or electron–phonon interactions, was increased from 0.00931 to 0.00943. The decrease in width of the localized tails of electronic states within the forbidden band gap of the donor can be expected from the decrease in the energy tail (E_tail_) and the increase in the σ value [31,32].

**Figure 4:**
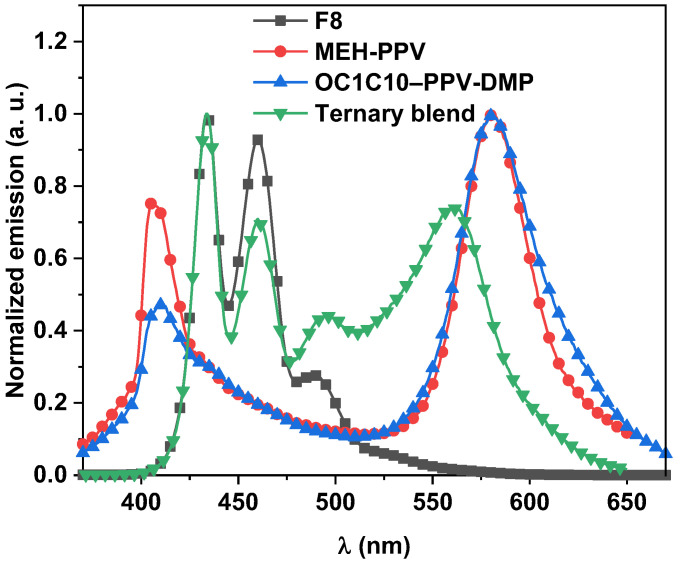
Normalized emission spectra of pristine F8, MEH-PPV, OC1C10-PPV-DMP, and their ternary hybrid.

The emission spectra of pristine thin films of F8, MEH-PPV, OC1C10-PPV-DMP and their optimal ternary hybrid at the excitation wavelength of 355 nm are shown in Figure 4. Two peaks at 434 nm and 460 nm and one shoulder at 492 nm (all placed in the blue region) were detected for F8, and two peaks observed in the violet region at 406 nm and 410 nm for MEH-PPV and OC1C10-PPV-DMP, respectively, in addition to another peak at 580 nm (yellow color) for both acceptors. By addition of these individual polymers to form the ternary blend, the emission was observed in the whole visible region and thus obtaining a white emission. Although the content of both acceptors was low (2 wt.%), their emission intensities in the yellow region were close to that of the donor. This result provides evidence for the FRET from F8 to both acceptors, where the mechanism of the dual FRET has been revealed in our recent report [27]. In the dual FRET mechanism, the excited molecules of F8 with light energy ~3.50 eV resulted in the generation of their oscillating dipoles and thus resonance with the dipoles of MEH-PPV and OC1C10-PPV-DMP molecules. Subsequently, the excited state energy transfers in space (without electron exchange) from the F8 molecules to the MEH-PPV molecules through dipole–dipole interaction. This type of transfer (Förster type) takes place when the F8 molecules return back to the ground state and the MEH-PPV molecules enter the excited state. Since identical processes take place for the OC1C10-PPV-DMP molecules, dual FRET mechanism can be carried out in the F8/MEH-PPV/OC1C10-PPV-DMP ternary hybrid thin-films.

**Figure 5:**
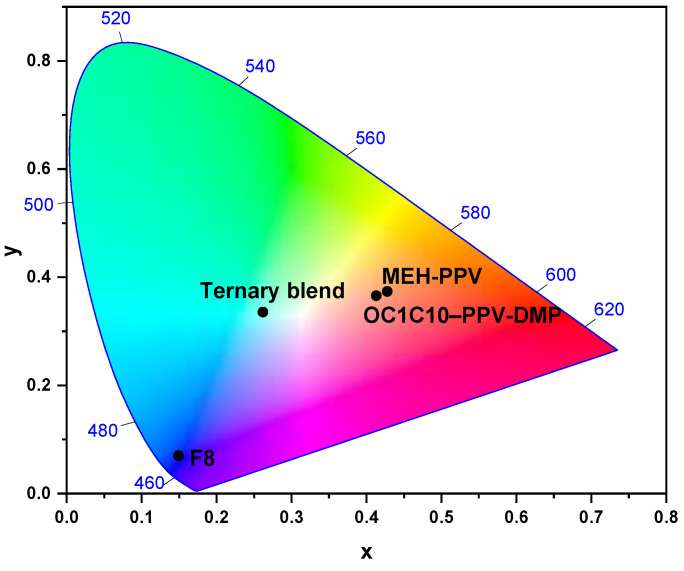
CIE coordinates for pristine F8, MEH-PPV, OC1C10–PPV-DMP, and their ternary hybrid.

The CIE coordinates of all pristine polymers and their ternary blend thin-films are displayed on the color chart of Figure 5. The CIE coordinates of pristine F8 were presented at the point (0.149, 0.067), confirming its blue emission, while they presented at the points (0.427, 0.374) and (0.413, 0.366) for MEH-PPV and OC1C10-PPV-DMP, respectively, confirming their existence on the whole visible region. The good white emission for the ternary hybrid was confirmed from their CIE coordinates at the point (0.262, 0.335). This observation reinforces our previous finding of an efficient dual Förster energy transfer in the ternary hybrid from F8 to both MEH-PPV and OC1C10-PPV-DMP.

**Figure 6:**
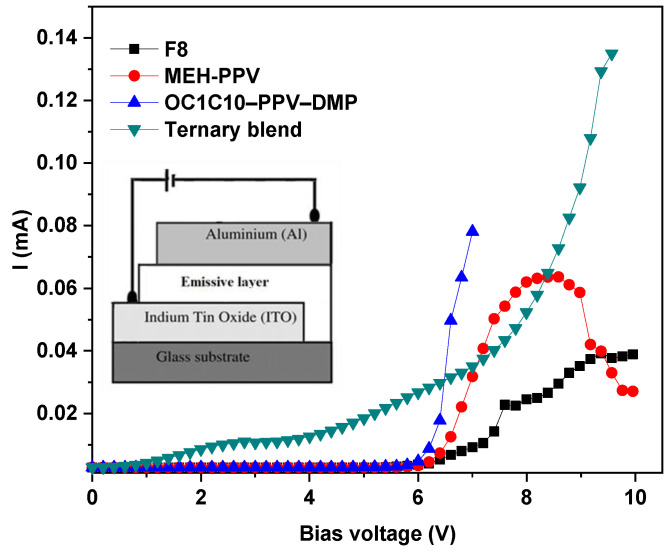
Current-voltage characteristics of OLEDs based on pristine F8, MEH-PPV, OC1C10-PPV-DMP, and their ternary hybrid. The inset is the schematic diagram of the OLED’s structure.

Figure 6 shows the current (I) under the forward bias (V) condition for the OLED devices based on pristine F8, MEH-PPV, OC1C10-PPV-DMP, and their ternary hybrid. Compared to the OLED devices based on pristine polymers, a significant increase in the current and a decrease in the turn-on voltage of injection current were revealed in the device based on ternary hybrid. The rise in the current as the direct result of the reduction in the resistance indicated the existence of a greater number of charge carriers in the emissive layer of the ternary hybrid and thus a lower turn-on voltage was achieved. These findings can be attributed to the existence carriers trapped by acceptors in addition to the dual FRET from F8 to both MEH-PPV and OC1C10-PPV-DMP, that resulted in an improvement of the device performance which is consistent with previous reports [24,27]. Since the HOMO and LUMO of each acceptor are located within that of F8 as shown in Figure 1b, the carriers (electrons and holes) can be trapped into the emissive layer of the device, resulting in increasing the exciton recombination. The dual FRET within the emissive layer of the device as well as the increased exciton recombination led together to raise the white emission and thus enhance the device performance.

## 4. Conclusions

The optoelectronic properties of the individual F8, MEH-PPV, and OC1C10-PPV-DMP can be successfully improved by blending them as a ternary blend. The existence of the narrow peaks that related to the semi-crystalline phase of the donor into the ternary hybrid indicated the good mixing of all of the conjugated polymers in the hybrid. Increasing the conjugation length and decreasing the width of the localized tails of electronic states within the forbidden band gap of the donor (F8) can be achieved by the addition of both acceptors, MEH-PPV and OC1C10-PPV-DMP, into the donor matrix with an appropriate ratio. This achievement was evidenced from the red-shifting of the main absorbance of F8 and decreasing its energy tail from 2.793 to 2.755 eV. The desired white emission can be obtained from the addition of the individual polymers in the form of ternary hybrid, as proved from its broadened emission spectrum in the whole visible region and its CIE coordinates at the point (0.262, 0.335). Moreover, the OLED based on the ternary blend exhibited a white emission with lower turn-on voltage compared to that based on the individual polymers, due to efficient charge trapping effect and FRET. The ternary hybrid of F8/2 wt.% MEH-PPV/2 wt.% OC1C10-PPV-DMP–based OLED with the addition of hole and electron transport layers will be very interesting for the design of high quality optoelectronic devices in future work.

## Data Availability

Not applicable.

## References

[1] Duan L., Zhang Y., Deng R., Yi H., Uddin A. (2020). Balance between energy transfer and exciton separation in ternary organic solar cells with two conjugated polymer donors. ACS Appl. Energy Mater..

[2] Sharma G., Kattayat S., Naqvi S.F., Hashmi S., Alvi P. (2021). Role of MEH: PPV polymer in single layer OLEDs with its optoelectronic characteristics. Mater. Today Proc..

[3] Ashizawa M., Zheng Y., Tran H., Bao Z. (2020). Intrinsically stretchable conjugated polymer semiconductors in field effect transistors. Prog. Polym. Sci..

[4] Mergu N., Kim H., Ryu J., Son Y.-A. (2020). A simple and fast responsive colorimetric moisture sensor based on symmetrical conjugated polymer. Sens. Actuator B Chem..

[5] Zhang S., Yue S., Wu Q., Zhang Z., Chen Y., Wang X., Liu Z., Xie G., Xue Q., Qu D. (2013). Color stable multilayer all-phosphor white organic light-emitting diodes with excellent color quality. Org. Electron..

[6] Divayana Y., Liu S., Kyaw A.K.K., Sun X.W. (2011). Efficient extraction of singlet–triplet excitons for high-efficient white organic light-emitting diode with a multilayer emission region. Org. Electron..

[7] Yang H., Xie W., Zhao Y., Hou J., Liu S. (2006). High efficiency small molecule white organic light-emitting devices with a multilayer structure. Solid State Commun..

[8] Liu J., Li W., Wang B., He Y., Miao T., Lü X., Fu G. (2020). Single-component white polymer light-emitting diode (WPLED) based on a binary tris-pyrazolonate-Sm-complex. J. Lumin..

[9] Gutiérrez–Llorente A. (2020). Effect of an assistant dopant on the vibrational satellites of a phosphorescent emitter: Application to solution–processed single–layer white organic light–emitting diodes. Org. Electron..

[10] da Silva M.A., Thomazini E.F., Albertini M., Renzi W., Franchello F., Dias I.F.L., Duarte J.L., Poças L.C., Lourenço S.A. (2016). Characterization of digital textile printing and polymer blend (PFO-DMP: P3HT) for application in manufacture of organic diodes emitting white light–WOLEDS. Opt. Mater..

[11] Mortensen K. (2015). Characterization of Polymer Blends Miscibility, Morphology and Interfaces.

[12] Förster T. (1982). Fluoreszenz Organischer Verbindungen.

[13] Allen N.S. (2010). Photochemistry and Photophysics of Polymeric Materials.

[14] Al-Asbahi B.A., Qaid S.M., Hj Jumali M.H., AlSalhi M.S., Aldwayyan A.S. (2019). Long-range dipole–dipole energy transfer enhancement via addition of SiO_2_/TiO_2_ nanocomposite in PFO/MEH-PPV hybrid thin films. J. Appl. Polym. Sci..

[15] Lakowicz J.R. (2006). Principles of Fluorescence Spectroscopy.

[16] Al-Asbahi B., Alsalhi M., Al-Dwayyan A., Jumali M.H. (2012). Förster-type energy transfer mechanism in PF2/6 to MEH-PPV conjugated polymers. J. Lumin..

[17] Virgili T., Lidzey D.G., Bradley D.D. (2000). Efficient energy transfer from blue to red in tetraphenylporphyrin-doped poly (9, 9-dioctylfluorene) light-emitting diodes. Adv. Mater..

[18] Cerullo G., Stagira S., Zavelani-Rossi M., De Silvestri S., Virgili T., Lidzey D.G., Bradley D.D.C. (2001). Ultrafast Förster transfer dynamics in tetraphenylporphyrin doped poly (9, 9-dioctylfluorene). Chem. Phys. Lett..

[19] Cossiello R.F., Susman M.D., Aramendía P.F., Atvars T.D. (2010). Study of solvent-conjugated polymer interactions by polarized spectroscopy: MEH–PPV and Poly (9, 9′-dioctylfluorene-2, 7-diyl). J. Lumin..

[20] List E.J., Creely C., Leising G., Graupner W. (2000). Excitation energy migration in highly emissive semiconducting polymers. Chem. Phys. Lett..

[21] Förster T. (1960). Transfer mechanisms of electronic excitation energy. Radiat. Res. Suppl..

[22] Shaheen S., Kippelen B., Peyghambarian N., Wang J.-F., Anderson J.D., Mash E.A., Lee P.A., Armstrong N.R., Kawabe Y. (1999). Energy and charge transfer in organic light-emitting diodes: A soluble quinacridone study. J. Appl. Phys..

[23] Mattoussi H., Murata H., Merritt C.D., Iizumi Y., Kido J., Kafafi Z.H. (1999). Photoluminescence quantum yield of pure and molecularly doped organic solid films. J. Appl. Phys..

[24] Al-Asbahi B.A. (2021). Dual Förster resonance energy transfer in ternary PFO/MEH-PPV/F7GA hybrid thin films for white organic light-emitting diodes. Dye. Pigm..

[25] Bajpai M., Srivastava R., Kamalasanan M., Tiwari R., Chand S. (2010). Charge transport and microstructure in PFO: MEH-PPV polymer blend thin films. Synth. Met..

[26] Al-Asbahi B.A., Haji Jumali M.H., AlSalhi M.S. (2016). Enhanced optoelectronic properties of PFO/Fluorol 7GA hybrid light emitting diodes via additions of TiO2 nanoparticles. Polymers.

[27] Al-Asbahi B.A., AlSalhi M.S., Fatehmulla A., Jumali M.H.H., Qaid S.M.H., Mujamammi W.M., Ghaithan H. (2021). Controlling the Emission Spectrum of Binary Emitting Polymer Hybrids by a Systematic Doping Strategy via Förster Resonance Energy Transfer for White Emission. Micromachines.

[28] Jumali M.H.H., Al-Asbahi B.A., Yap C.C., Salleh M.M., Alsalhi M.S. (2012). Optoelectronic property enhancement of conjugated polymer in poly (9, 9′-di-n-octylfluorenyl-2.7-diyl)/titania nanocomposites. Thin Solid Films.

[29] Sengwa R.J., Choudhary S. (2014). Structural characterization of hydrophilic polymer blends/montmorillonite clay nanocomposites. J. Appl. Polym. Sci..

[30] Zhang N., Li Z., Zhu C., Peng H., Zou Y. (2021). Bromination and increasing the molecular conjugation length of the non-fullerene small-molecule acceptor based on benzotriazole for efficient organic photovoltaics. RSC Adv..

[31] Keshav R., Padiyar M., Meghana N., Mahesha M. (2018). Analysis of PV deposited ZnTe thin films through Urbach tail and photoluminescence spectroscopy. J. Lumin..

[32] Qaid S.M., Al-Asbahi B., Ghaithan H.M., AlSalhi M. (2020). Optical and structural properties of CsPbBr_3_ perovskite quantum dots/PFO polymer composite thin films. J. Colloid Interface Sci..

